# Relationships Between *APOE*, Type 2 Diabetes, and Cardiovascular Disease in Postmenopausal Women

**DOI:** 10.1093/gerona/glae246

**Published:** 2024-10-04

**Authors:** Michelle M Dunk, Ira Driscoll, Mark A Espeland, Kathleen M Hayden, Simin Liu, Rami Nassir, Ginny Natale, Aladdin H Shadyab, JoAnn E Manson

**Affiliations:** Department of Psychology, University of Wisconsin–Milwaukee, Milwaukee, Wisconsin, USA; Department of Neurobiology, Aging Research Center, Care Sciences and Society, Karolinska Institutet, Stockholm, Sweden; Department of Psychology, University of Wisconsin–Milwaukee, Milwaukee, Wisconsin, USA; Alzheimer’s Disease Research Center, University of Wisconsin–Madison, Madison, Wisconsin, USA; Department of Biostatistics and Data Science, Wake Forest University School of Medicine, Winston-Salem, North Carolina, USA; Sticht Center for Healthy Aging and Alzheimer’s Prevention, Wake Forest University School of Medicine, Winston-Salem, North Carolina, USA; Division of Public Health Sciences, Department of Social Sciences and Health Policy, Wake Forest University School of Medicine, Winston-Salem, North Carolina, USA; Department of Epidemiology and Center for Global Cardiometabolic Health, School of Public Health, Brown University, Providence, Rhode Island, USA; Departments of Surgery and Medicine, The Warren Alpert Medical School, Brown University, Providence, Rhode Island, USA; Department of Pathology, School of Medicine, Umm Al-Qura University, Mecca, Saudi Arabia; Program in Public Health, Stony Brook University School of Medicine, Stony Brook, New York, USA; Hebert Wertheim School of Public Health and Human Longevity Science, University of California, San Diego, La Jolla, California, USA; Department of Medicine, Brigham and Women’s Hospital, Harvard Medical School, Boston, Massachusetts, USA; Department of Epidemiology, Harvard T.H. Chan School of Public Health, Boston, Massachusetts, USA; (Medical Sciences Section)

**Keywords:** Alzheimer’s disease, Apolipoprotein E ε4 allele, Cardiometabolic disease, Dementia, Genetic risk

## Abstract

**Background:**

The apolipoprotein E (*APOE*) ε4 allele, type 2 diabetes mellitus (T2DM), and cardiovascular disease (CVD) are well-established risk factors for dementia. Relationships between *APOE* and incidence of T2DM and CVD are not fully understood but may shed light on the mechanisms underlying dementia pathogenesis.

**Methods:**

Postmenopausal women (*N* = 6 795) from the Women’s Health Initiative hormone therapy clinical trial with *APOE* genotyping and no prior diagnosis of T2DM or CVD were included. We examined associations of *APOE* status (*APOE*2+ [ε2/ε2, ε2/ε3], *APOE*3 [ε3/ε3], and *APOE*4+ [ε4/ε4, ε3/ε4] carriers) with incidence of T2DM, coronary heart disease, stroke, and total CVD events using Cox regression. CVD outcomes were examined in baseline non-statin users and adjusted for statin initiation over follow-up to account for possible confounding by statins.

**Results:**

Among all participants (mean age 66.7 ± 6.5 years, 100% non-Hispanic White), 451 (6.6%) were using statins at baseline. Over the follow-up (mean 14.9 and 16.0 years for T2DM and CVD, respectively), 1 564 participants developed T2DM and 1 578 developed CVD. T2DM incidence did not differ significantly by *APOE* status (*p*s ≥ .09). Among non-statin users, *APOE*4+ had higher incidence of total CVD (hazard ratio [95% confidence interval] = 1.18 [1.02–1.38], *p* = .03) compared with *APOE*3 carriers, but risks for coronary heart disease (1.09 [0.87–1.36], *p* = .47) and stroke (1.14 [0.91–1.44], *p* = .27) were not significantly elevated when examined individually. CVD outcomes did not differ between *APOE*2+ and *APOE*3 carriers (*p*s ≥ 0.11).

**Conclusions:**

T2DM risk did not differ by *APOE* status among postmenopausal women, but *APOE*4+ carriers not using statins had an increased risk of total CVD events.

The apolipoprotein E (*APOE*) ε4 allele, type 2 diabetes mellitus (T2DM), and cardiovascular disease (CVD) each are established risk factors for dementia (1,2). T2DM and CVD are both associated with unfavorable changes in cognition and brain structure that likely contribute to this risk (3–5). Substantial evidence links dementia to cardiometabolic risk factors such as insulin resistance, glucose dysregulation, hypertension, dyslipidemia, and obesity, many of which are central aspects of T2DM and CVD (6,7). Specific mechanisms connecting *APOE* ε4 with cardiometabolic dysfunction and dementia, however, remain unclear. The global prevalence of both T2DM and CVD is projected to rise in the coming years (8,9), which could considerably impact dementia incidence. A better understanding of the relationships between *APOE*, T2DM, and CVD and their contributions to dementia risk is therefore critical for the development of more effective risk reduction strategies.

The *APOE* gene, located on chromosome 19, is the most potent genetic risk factor for Alzheimer’s disease (AD), the most common form of dementia (1). *APOE* plays a multifunctional role in lipid metabolism and brain physiology (10). Compared to non-ε4 allele carriers (*APOE*4−), carriers of one ε4 allele have a 2- to 4-fold increased risk of developing AD, while those with 2 ε4 alleles have an 8- to 12-fold greater risk (1,11). The ε3 allele appears neutral, while the ε2 allele has been associated with a lower risk of AD (1,11).

*APOE* has been implicated as a susceptibility locus for both T2DM and CVD, which could provide a potential link between cardiometabolic dysfunction and AD risk. *APOE*4 carriers tend to have an increased risk of CVD (12–14), although findings from epidemiological studies remain somewhat inconclusive (12–15). The literature on *APOE* and T2DM is also unclear, and prospective research on long-term T2DM incidence across *APOE* genotypes is limited. One meta-analysis of case–control studies suggests a higher prevalence of T2DM among *APOE*2 carriers (16), while a more recent meta-analysis reported a higher prevalence in ε2/ε2, ε3/ε4, and ε4/ε4 allele carriers (17). A prospective study of 436 dyslipidemia patients reported that T2DM incidence was higher only in *APOE*2 carriers (18).

Given *APOE*’s role in AD risk, a better understanding of its relationships with T2DM and CVD may clarify the contribution of cardiometabolic health to dementia risk and provide insights into prevention and treatment approaches. Here, we examine and clarify the relationships between *APOE* and incident T2DM and CVD in a large, well-characterized, prospective sample of postmenopausal women from the Women’s Health Initiative (WHI) clinical trial of hormone therapy (HT) (19).

## Method

### Participants

In the WHI HT, postmenopausal women age 50–79 were enrolled between 1993 and 1998 across 40 clinical centers in the United States and followed through 2021 (19). Participants were assigned to either active therapy of estrogen (0.625 mg/day of conjugated equine estrogens [CEE]) if prior hysterectomy, estrogen plus progestin (0.625 mg/day of CEE + 2.5 mg/day of medroxyprogesterone acetate) if no prior hysterectomy, or matching placebo (19). The estrogen and estrogen plus progestin trials continued for 5.6 and 7.2 years, respectively (20). The current sample included 6 795 WHI HT participants with no prior history of T2DM, CVD (myocardial infarction or stroke), or other vascular conditions or events (cardiac arrest, congestive heart failure, transient ischemic attack, coronary bypass surgery, angioplasty of coronary arteries, cardiac catheterization, carotid endarterectomy or angioplasty, peripheral arterial disease, atrial fibrillation, aortic aneurysm, or angina) who were genotyped for *APOE* and had available serum lipids measured from baseline blood draw. A total of 625 women were excluded due to prevalent T2DM (100 [16%] *APOE*2+, 400 [64%] *APOE*3, 125 [20%] *APOE*4+), and 1 507 were excluded due to prevalent CVD (199 [13%] *APOE*2+, 957 [64%] *APOE*3, 351 [23%] *APOE*4+) at baseline. All participants provided written informed consent and study approval was obtained by the National Institutes of Health and the Institutional Review Boards of participating sites.

Baseline demographic and medical information collected via self-report or standardized assessments included age, education (less than high school, high school graduate, some college, college graduate, or postgraduate education), annual family income (<$20 000, $20 000 to <$50 000, $50 000 to <$100 000, or ≥$100 000), body mass index (BMI; kg/m^2^), physical activity (metabolic equivalent of task hours per week), alcohol consumption (number of servings per week of beer, wine, and liquor based on a medium serving size [12 ounces of beer, 6 ounces of wine, 1.5 ounces of liquor]), self-reported hypertension (yes or no), and smoking status (never, former, or current) (19). Serum lipids (low-density lipoprotein [LDL] and high-density lipoprotein [HDL] cholesterol [mg/dL] and triglycerides [mg/dL]) were measured from blood drawn at baseline (19). Fasting serum glucose (mmol/L) and insulin (μU/L) were also measured from baseline blood draw, from which insulin resistance was determined using the homeostatic model assessment of insulin resistance (HOMA-IR) by multiplying insulin and glucose levels and dividing by 22.5 (21,22). Statin therapy was assessed at baseline and years 1, 3, 6, and 9 throughout follow-up, as well as Year 5 of the first WHI Extension Study (19).

### *APOE* Genotyping

*APOE* genotype was determined from baseline blood samples according to SNPs rs429358 and rs7412. Genotyping was based on imputation and harmonization of genetic data across 2 WHI genome-wide association studies (GWAS), the Genomics and Randomized Trials Network (GARNET) and WHI Memory Study+ (WHIMS+). The GWAS platforms used for GARNET and WHIMS+ were the Illumina HumanOmni1-Quad v1-0 B and HumanOmniExpressExome 8v1_B, respectively, and the 1000 Genomes Project reference panel was used for imputation in both GWAS studies (23). We classified participants into 3 groups based on the 6 common *APOE* genotypes: *APOE*2+ (ε2/ε2, ε2/ε3), *APOE*3 (ε3/ε3), and *APOE*4+ (ε4/ε4, ε3/ε4) carriers. As in many other studies of this nature, those with ε2/ε4 genotype (*n* = 172) were excluded from analyses because the ε2 and ε4 alleles are proposed to have opposing effects on CHD risk (14,15), making it unclear whether they should be grouped with *APOE*2+ or *APOE*4+ carriers.

### Assessment of Disease Endpoints

#### Type 2 diabetes mellitus

Incident T2DM during follow-up was defined as a self-report of a new T2DM diagnosis by a physician which was treated with insulin or oral medication (24). Self-reported incidence was compared with, and consistent with, medication inventories and fasting glucose levels throughout follow-up (25).

#### Cardiovascular disease

Three CVD endpoints—coronary heart disease (CHD) composite, stroke composite, and total CVD—were examined to determine whether *APOE* relates more to CHD, stroke, or overall CVD outcomes. CHD composite included nonfatal myocardial infarction and death from CHD. Stroke composite included nonfatal or fatal stroke. Total CVD included CHD and stroke composites as well as total CVD death (death due to CHD, cerebrovascular causes, pulmonary embolism, or other cardiovascular causes). CVD outcomes were adjudicated by trained physicians using previously published standard criteria (26). WHI reviewed all hospitalization records for CVD-related outcomes during the HT intervention phase and ascertained deaths using records obtained from periodic searches of the National Death Index to complement routine follow-up reports of deaths by next of kin and postal authorities (26).

### Statistical Analysis

Linear regression was performed to examine differences in cardiometabolic measurements (glucose, insulin, HOMA-IR, LDL and HDL cholesterol, and triglycerides) by *APOE* status (*APOE*3 as reference) while adjusting for age, BMI, smoking status, alcohol intake, education, income, HT assignment, and statin therapy at baseline. Relationships between *APOE* status and disease outcomes were investigated using Cox proportional hazards regression, with *APOE*3 as the reference group. Associations between *APOE* and T2DM were initially analyzed in an unadjusted model, followed by adjustment for baseline age, BMI, alcohol intake, smoking status, education, income, HT assignment, hypertension, and statin therapy. To account for possible confounding by statin use at baseline and throughout follow-up (27), associations between *APOE* and CVD outcomes were examined in baseline non-statin users (*N* = 6 344), and those who initiated statin therapy during follow-up were right-censored at that date if they had not yet experienced a CVD event. *APOE*-CVD associations were first analyzed in an unadjusted model, followed by adjustment for baseline age, BMI, alcohol intake, smoking status, education, income, HT assignment, hypertension, and T2DM incidence throughout follow-up.

#### Sensitivity analysis

Sensitivity analyses of T2DM incidence were performed (1) after adjusting for LDL cholesterol, HDL cholesterol, and triglycerides at baseline and (2) after excluding participants using statins at baseline (*n* = 451) and accounting for statin use throughout follow-up by right-censoring at the date of statin initiation. We similarly examined CVD incidence (1) after controlling for blood lipid levels at baseline and (2) among the entire sample (including baseline statin users), followed by stratified and interaction analyses according to baseline statin use. We also examined baseline prevalence of T2DM and CVD (myocardial infarction, stroke, or total CVD events) by *APOE* after including all participants previously excluded due to those baseline diagnoses (*n* = 1 949) using logistic regression (total *N* = 8 744).

Analyses were performed using SAS Studio on the SAS OnDemand for Academics platform, version 3.8 (Copyright 2012–2018, SAS Institute Inc., Cary, NC).

## Results

### Sample Characteristics

Characteristics of participants at WHI enrollment are presented in Table 1. The sample included 912 (13%) *APOE*2+, 4,271 (63%) *APOE*3, and 1 612 (24%) *APOE*4+ carriers. The mean age at enrollment was 66.7 (*SD* = 6.5) years, and all women were non-Hispanic White. For purposes of generalization, this sample’s demographics resemble those of the larger WHI HT aside from having an older average age at enrollment by approximately 3 years and being restricted by race/ethnicity (20). Participants were followed up to 26 years, between 1993 and 2021. After an average of 14.9 (standard deviation [*SD*] = 7.0) years, 1 564 (23%) women were diagnosed with T2DM during follow-up. After an average of 16 (*SD* = 6.8) years, 1 578 incident cases (23%) of total CVD were documented. A total of 768 (11%) CHD composite events took place after an average of 16.4 (*SD* = 6.6) years, and 686 (10%) stroke composite cases occurred following a mean of 16.5 (*SD* = 6.6) years. At baseline, 1 967 (28.9%) participants had hypertension. Statin therapy at baseline and statin initiation during follow-up were more common among *APOE*3 and *APOE*4+ compared with *APOE*2+ carriers, with 451 (6.6%) participants in total using statin medication at baseline and 2 347 (34.5%) subsequently beginning statin therapy. There were no differences in fasting glucose, insulin, or HOMA-IR by *APOE* groups, but *APOE*4+ did have higher LDL cholesterol and triglycerides and lower HDL cholesterol, while *APOE*2+ had lower LDL and higher HDL cholesterol compared with *APOE*3 (Supplementary Table 1).

**Table 1.** Participant Characteristics at Baseline by *APOE* Status

Characteristic	Pooled, *N* = 6 795	*APOE*2+, *N* = 912	*APOE*3, *N* = 4 271	*APOE*4+, *N* = 1 612	*p* Value
Age, y	66.7 ± 6.5	67.0 ± 6.7	66.7 ± 6.5	66.4 ± 6.4	0.07
Education					0.56
Less than HS graduation	315 (4.6)	41 (4.5)	206 (4.8)	68 (4.2)	
HS graduate	1 482 (21.8)	219 (24.0)	919 (21.5)	344 (21.3)	
Some college	2 718 (40.0)	349 (38.3)	1 718 (40.2)	651 (40.4)	
College graduate	670 (9.9)	89 (9.8)	424 (9.9)	157 (9.7)	
Postgraduate education	1 588 (23.4)	213 (23.4)	987 (23.1)	388 (24.1)	
Family income, USD					0.38
<20 000	1 309 (19.3)	162 (17.8)	818 (19.2)	329 (20.4)	
20 000–49 999	3 346 (49.2)	455 (49.9)	2 122 (49.7)	769 (47.7)	
50 000–99 999	1 466 (21.6)	212 (23.2)	890 (20.8)	364 (22.6)	
≥100 000	310 (4.6)	38 (4.2)	198 (4.6)	74 (4.6)	
HRT					0.60
CEE intervention	1 212 (17.8)	151 (16.6)	784 (18.4)	277 (17.2)	
CEE control	1 240 (18.2)	154 (16.9)	793 (18.6)	293 (18.2)	
CEE + MPA intervention	2 219 (32.7)	331 (36.3)	1 339 (31.4)	549 (34.1)	
CEE + MPA control	2 124 (31.3)	276 (30.3)	1 355 (31.7)	493 (30.6)	
BMI, kg/m^2^	28.6 ± 5.8	28.5 ± 5.4	28.7 ± 5.8	28.3 ± 5.9	0.05
Physical activity (MET hrs/wk)	11.6 ± 13.2	11.7 ± 14.0	11.6 ± 13.0	11.6 ± 13.3	0.97
Smoking status					0.55
Never	3 476 (51.2)	450 (49.3)	2 220 (52.0)	806 (50.0)	
Former	2 672 (39.3)	360 (39.5)	1 674 (39.2)	638 (39.6)	
Current	578 (8.5)	93 (10.2)	335 (7.8)	150 (9.3)	
Weekly alcohol intake	2.7 ± 5.6	2.7 ± 5.6	2.8 ± 5.6	2.7 ± 5.4	0.91
Glucose, mmol/L	5.4 ± 1.1	5.4 ± 1.1	5.4 ± 1.2	5.4 ± 1.1	0.60
Insulin, μU/L	9.2 ± 6.8	9.0 ± 5.9	9.3 ± 7.0	9.1 ± 6.9	0.40
HOMA-IR	2.3 ± 2.2	2.3 ± 1.8	2.4 ± 2.3	2.3 ± 2.3	0.34
Statin therapy at baseline	451 (6.6)	29 (3.2)	288 (6.7)	134 (8.3)	<0.001
Statin initiation over follow-up	2 347 (34.5)	222 (24.3)	1 531 (35.8)	594 (36.8)	<0.001
Hypertension	1 967 (28.9)	257 (28.2)	1 229 (28.8)	481 (29.8)	0.63
HDL cholesterol, mg/dL	53.4 ± 12.4	55.3 ± 12.8	53.5 ± 12.1	52.0 ± 12.7	<0.001
LDL cholesterol, mg/dL	154.5 ± 35.6	135.8 ± 33.1	156.0 ± 34.7	161.0 ± 35.9	<0.001
Triglycerides, mg/dL	138.8 ± 66.1	137.2 ± 61.8	137.0 ± 65.3	144.4 ± 70.2	<0.001

*Notes*: Values are presented as *N* (%) or *M* ± SD. Glucose and insulin, measured in serum from blood draw following at least 8 hours of fasting, were used to calculate HOMA-IR according to the equation: [insulin (μU/L)×glucose (mmol/L)]/22.5 (21, 22). Missing data: Education = 22; family income = 364; BMI = 41; physical activity = 406; smoking status = 69; weekly alcohol intake = 27; glucose = 15; insulin = 256; HOMA-IR = 256. *APOE* = Apolipoprotein E; BMI = body mass index; CEE = conjugated equine estrogen; HDL = high-density lipoprotein; HOMA-IR = homeostatic model assessment of insulin resistance; HRT = hormone replacement therapy; HS = high school; LDL = low-density lipoprotein; M = mean; MET = metabolic equivalent of task; MPA = medroxyprogesterone acetate; SD = standard deviation.

### Type 2 Diabetes Mellitus

**Figure 1.**
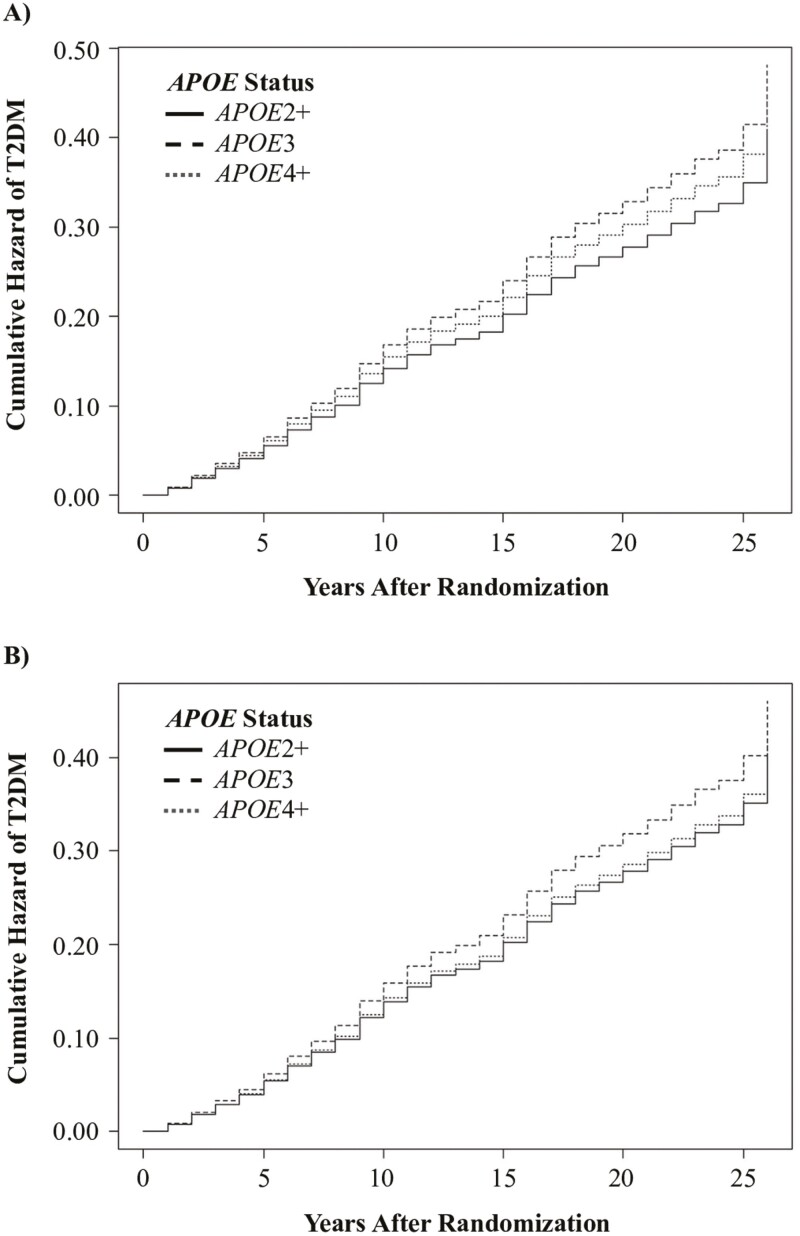
Cumulative hazard curves from Cox regression depicting (**A**) unadjusted type 2 diabetes mellitus (T2DM) incidence and (**B**) adjusted T2DM incidence by apolipoprotein E (*APOE*) status.

In unadjusted analysis, T2DM incidence was significantly lower in *APOE*2+ compared with *APOE*3 carriers (hazard ratio = 0.85 [95% confidence interval (CI): 0.73–0.99], *p* = .03; Table 2) and did not differ significantly between *APOE*4+ and *APOE*3 carriers (0.92 [0.82–1.04], *p* = .20). In adjusted analysis, T2DM incidence did not differ in *APOE*2+ (0.87 [0.75–1.03], *p* = .10) or *APOE*4+ (0.90 [0.79–1.02], *p* = .10) compared with *APOE*3 carriers. Cumulative hazard rates for T2DM incidence based on *APOE* status are presented in Figure 1.

**Table 2.** Associations Between *APOE* and T2DM Incidence

*APOE*	Cases/Total	Unadjusted Model		Adjusted Model	
		HR (95% CI)	*p* Value	HR (95% CI)	*p* Value
*APOE*2+	192/912	0.85 (0.73–0.99)	0.03	0.87 (0.75–1.03)	0.10
*APOE*3	1 027/4 271	Reference		Reference	
*APOE*4+	345/1 612	0.92 (0.82–1.04)	0.20	0.90 (0.79–1.02)	0.10

*Notes*: The adjusted model controlled for age, BMI, education, income, smoking status, alcohol intake, hormone therapy, hypertension, and statin therapy at baseline. *APOE* = apolipoprotein E; BMI = body mass index; CI = confidence interval; HR = hazard ratio; T2DM = type 2 diabetes mellitus.

#### Sensitivity analysis

After further adjusting for baseline lipids, T2DM incidence was lower in *APOE*4+ compared with *APOE*3 carriers (0.84 [0.74–0.95], *p* = .007; Supplementary Table 2). Incidence of T2DM did not differ by *APOE* status among baseline nonusers of statins in adjusted analysis (Supplementary Table 3). Baseline prevalence of T2DM was marginally lower in *APOE*4+ (0.81 [0.64–1.01], *p* = .06) but did not differ between *APOE*2+ (1.24 [0.97–1.59], *p* = .09) and *APOE*3 carriers (Supplementary Table 4).

### Cardiovascular Disease

**Figure 2.**
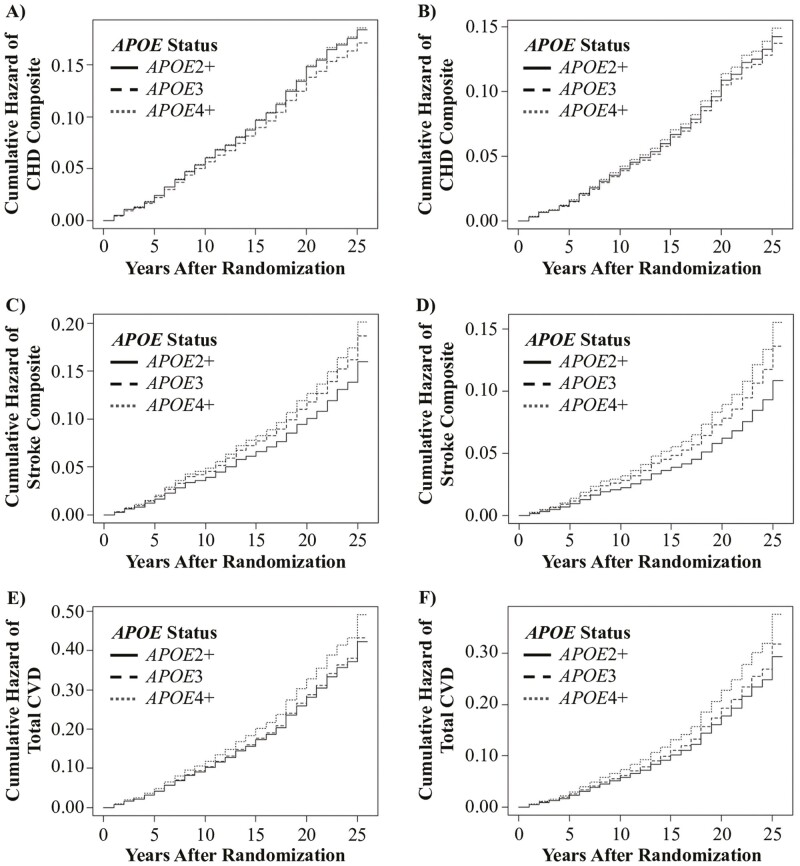
Cumulative hazard curves from Cox regression depicting incidence of (**A**) unadjusted coronary heart disease (CHD) composite, (**B**) adjusted CHD composite, (**C**) unadjusted stroke composite, (**D**) adjusted stroke composite, (**E**) unadjusted total cardiovascular disease (CVD), and (**F**) adjusted total CVD events by apolipoprotein E (*APOE*) status.

Among baseline nonusers of statins and after censoring at the time of statin initiation throughout follow-up, *APOE*4+ carriers had significantly higher incidence of total CVD (1.18 [1.02–1.38], *p* = .03), though not CHD composite (1.09 [0.87–1.36], *p* = .47) or stroke composite (1.14 [0.91–1.44], *p* = .27), compared with *APOE*3 carriers in adjusted analysis (Table 3). In *APOE*2+ carriers, neither CHD composite (1.04 [0.81–1.33], *p* = .78), stroke composite (0.80 [0.60–1.06], *p* = .11), nor total CVD events (0.92 [0.77–1.10], *p* = .38) differed significantly compared with *APOE*3 carriers. Cumulative hazard rates of all 3 CVD outcomes by *APOE* status are displayed in Figure 2.

**Table 3.** CVD Incidence Among Baseline Non-Statin Users (*N* = 6 344) Accounting for Statin Initiation During Follow-Up

CVD Outcome	Cases/Total	Unadjusted Model		Adjusted Model	
		HR (95% CI)	*p* Value	HR (95% CI)	*p* Value
CHD composite					
* APOE*2+	88/883	1.07 (0.85–1.36)	0.56	1.04 (0.81–1.33)	0.78
* APOE*3	319/3 983	Reference		Reference	
* APOE*4+	118/1 478	1.08 (0.88–1.34)	0.46	1.09 (0.87–1.36)	0.47
Stroke composite					
* APOE*2+	65/883	0.86 (0.65–1.12)	0.26	0.80 (0.60–1.06)	0.11
* APOE*3	289/3 983	Reference		Reference	
* APOE*4+	105/1 478	1.08 (0.86–1.35)	0.52	1.14 (0.91–1.44)	0.27
Total CVD					
* APOE*2+	168/883	0.98 (0.83–1.16)	0.80	0.92 (0.77–1.10)	0.38
* APOE*3	657/3 983	Reference		Reference	
* APOE*4+	251/1 478	1.14 (0.98–1.32)	0.08	1.18 (1.02–1.38)	0.03

*Notes*: The adjusted model controlled for age, BMI, education, income, smoking status, alcohol intake, hormone therapy, and hypertension at baseline as well as T2DM incidence throughout follow-up. *APOE* = Apolipoprotein E; BMI = body mass index; CHD = coronary heart disease; CI = confidence interval; CVD = cardiovascular disease; HR = hazard ratio.

#### Sensitivity analysis

CVD incidence among non-statin users did not differ significantly by *APOE* status after adjustment for baseline blood lipid levels (Supplementary Table 5). In the analysis of the entire sample, CVD incidence did not differ by *APOE* status (Supplementary Table 6). There was a significant interaction between *APOE* and baseline statin therapy in relation to CHD composite and total CVD events, whereby *APOE*4+ carriers using statins had significantly lower risk (*p*s for interactions = 0.03) compared with *APOE*3 carriers not using statins (Supplementary Figure 1). *APOE*2+ carriers using statins had higher total CVD risk compared to *APOE*3 carriers not using statins (*p* for interaction = 0.04). In stratified analysis, among nonusers of statins, *APOE*4+ carriers had higher total CVD risk (1.15 [1.01–1.31], *p* = .04) compared with *APOE*3 carriers. Among statin users, *APOE*4+ carriers had lower CHD risk compared with *APOE*3 carriers (Supplementary Figure 1). Prevalence of myocardial infarction, stroke, and total CVD at baseline did not differ significantly by *APOE* status (*p*s ≥ 0.12; Supplementary Table 7).

## Discussion

In this large prospective cohort of non-Hispanic white postmenopausal women, we report (1) no significant differences in T2DM incidence by *APOE* status and (2) higher total CVD incidence in *APOE*4+ compared with *APOE*3 carriers among non-statin users. Our results are in line with previous associations between *APOE* and CVD outcomes but diverge from some of the literature regarding associations between *APOE* and T2DM risk.

Existing reports on *APOE*’s association with T2DM are conflicting, and the majority of studies have examined T2DM prevalence rather than incidence. A meta-analysis of 30 case–control studies reported a moderate association between *APOE*2 and T2DM risk (16). A separate meta-analysis of 59 case–control studies reported that the *APOE* ε4 allele and ε2/ε2, ε3/ε4, and ε4/ε4 genotypes were more common among T2DM patients (17). In a more recent case–control study, T2DM patients were more likely to be *APOE*4+ or have ε3/ε4 genotype compared to controls, especially in those with CHD or cerebral infarction, while the ε2/ε3 genotype was more common in T2DM patients with diabetic nephropathy (28). Among a sample of 436 patients with dyslipidemia or suspected familial dyslipidemia, *APOE*2+ carriers had the highest incidence of T2DM (18). A large-scale case–control, phenome-wide association study in the UK Biobank reported lower T2DM risk among ε4/ε4 and ε3/ε4 carriers (29). However, another phenome-wide association study in the UK Biobank observed no difference in combined prevalence and incidence of T2DM in those with ε2/ε2 or ε2/ε3 compared to ε3/ε3 genotype (30).

We found no differences in T2DM incidence by *APOE* status, which remained even after censoring by statin initiation throughout follow-up. While this finding is consistent with the investigation by Kuo et al. (30), our results could also be impacted by our exclusively female sample, in contrast to the previous studies which included both males and females (16–18,28). Males tend to have a higher prevalence of T2DM, whereas females ≥60 years of age have a higher prevalence of undiagnosed T2DM (31).

The higher incidence of total CVD in *APOE*4+ carriers not using statins in our sample is largely consistent with prior studies, which tend to report increased CVD risk in relation to *APOE*4 (13,14,29). Interestingly, while Scuteri et al. reported that *APOE*4 was a risk factor for coronary events in men but not women (12), our exclusively female sample did reveal an association between *APOE*4 and total CVD in nonusers of statins. The mean ages of the current sample and the sample in the study by Scuteri et al. were 66.7 and 52, respectively, so the age difference could have influenced these opposing findings (12).

*APOE*’s known influence on lipid metabolism could be one of the mechanisms underlying *APOE*’s involvement in CVD and T2DM. The *APOE* ε4 allele is associated with higher total and LDL cholesterol, whereas the ε2 allele is associated with lower levels of these lipids (13,14). Consequently, *APOE*4 carriers have a higher prevalence of dyslipidemia (13,32), a major risk factor for CVD (7). The elevated risk of total CVD in *APOE*4+ carriers disappeared after controlling for LDL and HDL cholesterol and triglycerides, and we also identified distinct variations in the association between *APOE*4+ status and CVD incidence according to baseline statin use. Both findings underscore the role of dyslipidemia in *APOE*-related CVD development. While *APOE*4+ carriers not using statins had elevated CVD risk, this risk was reversed among those using statins, signifying that *APOE*4-related CVD risk may be modifiable through lipid management.

Higher total cholesterol is also associated with decreased insulin secretory capacity (33) and impaired function of pancreatic beta cells (34), both core features of T2DM (6). Notably, we observed lower T2DM incidence among *APOE*4+ carriers after adjusting for blood lipids, which could indicate a mechanistic link between *APOE*-related dyslipidemia and T2DM risk. In contrast to *APOE* ε4-associated dyslipidemia, *APOE*2 carriers tend to have high triglyceride levels (14,35), and up to 10% of *APOE*2 homozygotes have familial dysbetalipoproteinemia, an atherogenic disorder characterized by an accumulation of triglyceride-rich lipoprotein remnants (36). Elevated triglycerides are a risk factor for T2DM (37) and are linked to hyperinsulinemia, especially in the presence of risk factors such as inflammation and adiposity related to a poor diet (36,38). Indeed, several studies have reported higher T2DM risk among *APOE*2+ carriers (16–18). Yet *APOE*2+ carriers in our sample did not have higher baseline triglycerides compared with *APOE*3, which could explain why we did not observe higher T2DM incidence in this group. These relationships between *APOE*, lipids, CVD, and T2DM suggest a plausible pathway through which *APOE* affects CVD and T2DM risks. However, inconsistencies in these associations in the existing literature may be obscured due to higher statin use among *APOE*4 carriers and higher T2DM risk associated with statin therapy (39).

In addition to *APOE*’s involvement in lipid metabolism, *APOE*4 is associated with hypertension (40) and obesity (41), both of which are risk factors for CVD and T2DM (42,43). Both *APOE*4 carriers and T2DM patients also have an elevated risk for atherosclerosis (44,45), the primary cause of CVD (46). CVD is estimated to affect 32% of T2DM patients (47), and CVD risk increases with higher fasting blood glucose levels and greater insulin resistance even in those without a history of diabetes (48,49). Moreover, CVD is a major cause of death among T2DM patients (47). In light of *APOE*4’s fairly consistent associations with CVD and its risk factors, the connections between CVD and T2DM insinuate, by extension, *APOE*’s involvement in T2DM.

Considering the substantial risk of AD in *APOE*4 carriers, connections between *APOE*, T2DM, and CVD may implicate cardiometabolic dysfunction as an integral component of AD. Indeed, T2DM, CVD, and related risk factors—namely, midlife hypercholesterolemia, hypertension, obesity, and poor glycemic control—are all associated with greater dementia risk (2,7). While *APOE*2 carriers appear to be protected against AD despite their suggested T2DM risk in some of the literature (16–18), maintenance of cardiometabolic health may be especially helpful for reducing the elevated AD risk among *APOE*4 carriers—those at greatest risk for AD—given their heightened risk of CVD (13,14). An estimated 40% of dementia cases worldwide could potentially be prevented or delayed through the management of cardiometabolic risk factors such as diet, physical activity, and avoidance of alcohol and smoking (2). *APOE*4 carriers appear to be more susceptible to lifestyle-related risk factors (50,51), highlighting the importance of managing cardiometabolic health in these individuals to mitigate AD risk.

One limitation of this study is the reliance on participant self-report of physician-prescribed insulin or oral antidiabetic medication for ascertainment of T2DM incidence (33). T2DM cases that were only being treated using lifestyle interventions would have gone unnoticed which, in combination with other undiagnosed cases, could have resulted in an underestimation of T2DM incidence (25). The reliability of self-reported T2DM incidence also could have varied across *APOE* groups. However, the majority of self-reported T2DM cases in WHI were consistent with the medication inventory and fasting glucose levels at baseline (25). Our findings are also limited to postmenopausal non-Hispanic White women due to the availability of genetic data and cannot be generalized to men or younger women. Strengths of our study also warrant consideration, including a large sample of well-screened and prospectively followed older women who underwent annual follow-up visits. Additionally, given the inconsistencies between previous reports of CVD risk based on *APOE* status as well as the lack of long-term studies examining *APOE*-related T2DM incidence, our study offers a unique contribution to the literature.

In conclusion, we observed associations between *APOE* genotype and incidence of CVD, but not T2DM in postmenopausal, non-Hispanic White women. The prevalence of statin use at baseline was higher among women with *APOE*4+ status, and the rate of statin initiation during follow-up was higher among *APOE*3 and *APOE*4+ carriers. When accounting for these differences in analyses among women not using statins at baseline, *APOE*4+ carriers had a higher incidence of total CVD events. Future research is needed to illuminate the specific mechanisms by which *APOE* relates to T2DM and CVD risks, and also how *APOE*-related susceptibility to these cardiometabolic conditions might contribute to subsequent dementia risk.

## Supplementary Material

glae246_suppl_Supplementary_Material

glae246_suppl_Supplementary_Appendix

## Data Availability

Data used for the present study are the property of the National Institutes of Health. Copies of the de-identified data used in our study can be made available upon request to, and pending approval by, the Women’s Health Initiative Publications and Presentations Committee (email: p&p@WHI.org).
