# Supplementary material for: Relationships Between APOE, Type 2 Diabetes, and Cardiovascular Disease in Postmenopausal Women
Source: J Gerontol A Biol Sci Med Sci. 2024 Oct 4;80(2):glae246. doi: 10.1093/gerona/glae246 (PMC11775828; doi:10.1093/gerona/glae246)
Supplement: glae246_suppl_Supplementary_Material [file glae246_suppl_supplementary_material.pdf]

Supplementary Material

Supplementary Table 1. Cardiometabolic profile by *APOE* status.

Supplementary Table 2. T2DM incidence based on *APOE* status after adjusting for blood lipids.

Supplementary Table 3. T2DM incidence among baseline non-statin users (N=6,344), accounting for statin initiation during follow-up.

Supplementary Table 4. Baseline prevalence of T2DM based on *APOE* status (N=8,744).

Supplementary Table 5. CVD incidence based on *APOE* status after adjusting for blood lipids.

Supplementary Table 6. CVD incidence based on *APOE* status, including statin users at baseline (N=6,795).

Supplementary Figure 1. CVD incidence based on *APOE* status, according to baseline statin therapy (N=6,795).

Supplementary Table 7. Baseline prevalence of CVD based on *APOE* status (N=8,744).

Supplementary Table 1. Cardiometabolic profile by *APOE* status.

Cardiometabolic profile	N	LSM ± SE	β (95% CI)	*p*-value
**Glucose (mmol/L)**				
*APOE*2+	909	5.44 ± 0.05	-0.02 (-0.10, 0.06)	0.64
*APOE*3	4,265	5.46 ± 0.03	Reference	
*APOE*4+	1,606	5.45 ± 0.04	-0.01 (-0.07, 0.06)	0.77
**Insulin (μU/L)**				
*APOE*2+	872	9.32 ±0.27	-0.21 (-0.66, 0.24)	0.37
*APOE*3	4,127	9.53 ± 0.19	Reference	
*APOE*4+	1,540	9.50 ± 0.22	-0.03 (-0.39, 0.33)	0.86
**HOMA-IR**				
*APOE*2+	872	2.33 ± 0.09	-0.09 (-0.24, 0.06)	0.23
*APOE*3	4,127	2.42 ± 0.06	Reference	
*APOE*4+	1,540	2.41 ± 0.07	-0.01 (-0.13, 0.11)	0.86
**LDL cholesterol (mg/dL)**				
*APOE*2+	912	132.41 ± 1.52	-20.43 (-23.01, -17.85)	<0.0001
*APOE*3	4,271	152.84 ± 1.05	Reference	
*APOE*4+	1,612	157.67 ± 1.24	4.82 (2.77, 6.88)	<0.0001
**HDL cholesterol (mg/dL)**				
*APOE*2+	912	52.98 ± 0.49	1.53 (0.70, 2.36)	0.0003
*APOE*3	4,271	51.45 ± 0.34	Reference	
*APOE*4+	1,612	49.55 ± 0.40	-1.90 (-2.57, -1.24)	<0.0001
**Triglycerides (mg/dL)**				
*APOE*2+	912	154.77 ± 2.76	1.25 (-3.43, 5.93)	0.60
*APOE*3	4,271	153.52 ± 1.91	Reference	
*APOE*4+	1,612	162.07 ± 2.26	8.55 (4.82, 12.29)	<0.0001

The models controlled for age, BMI, education, income, smoking status, alcohol intake, hormone therapy, and statin therapy at baseline.
Missing data: glucose, n=15; HOMA-IR, n=256; insulin, n=256.
Abbreviations: APOE=Apolipoprotein E; BMI=body mass index; CI=confidence interval; HDL=high-density lipoprotein; LDL=low-density lipoprotein; LSM=least-squares mean; SE=standard error.

Supplementary Table 2. T2DM incidence based on *APOE* status after adjusting for blood lipids.

APOE	**Cases/Total**	**HR (95% CI)**	***p*-value**
*APOE*2+	192/912	0.89 (0.75-1.05)	0.15
*APOE*3	1,027/4,271	Reference	
*APOE*4+	345/1,612	0.84 (0.74-0.95)	0.007

The model controlled for age, BMI, education, income, smoking status, alcohol intake, hormone therapy, hypertension, statin therapy, triglycerides, LDL cholesterol, and HDL cholesterol at baseline.
Abbreviations: *APOE*=Apolipoprotein E; BMI=body mass index; CI=confidence interval; HR=hazard ratio; T2DM=type 2 diabetes mellitus.

Supplementary Table 3. T2DM incidence among baseline non-statin users (N=6,344), accounting for statin initiation during follow-up.

	**Prevalent**	**Unadjusted Model**		**Adjusted Model**	
APOE	**Cases/Total**	**HR (95% CI)**	***p*-value**	**HR (95% CI)**	***p*-value**
*APOE*2+	156/883	0.80 (0.67-0.95)	0.01	0.86 (0.72-1.02)	0.09
*APOE*3	748/3,983	Reference		Reference	
*APOE*4+	228/1,478	0.87 (0.75-1.01)	0.07	0.87 (0.75-1.02)	0.08

The adjusted model controlled for age, BMI, education, income, smoking status, alcohol intake, hormone therapy, and hypertension at baseline.
Abbreviations: *APOE*=Apolipoprotein E; BMI=body mass index; CI=confidence interval; HR=hazard ratio; T2DM=type 2 diabetes mellitus.

Supplementary Table 4. Baseline prevalence of T2DM based on *APOE* status (N=8,744).

	Prevalent	Unadjusted Model		Adjusted Model	
APOE	**Cases/Total**	**OR (95% CI)**	***p*-value**	**OR (95% CI)**	***p*-value**
*APOE*2+	100/1,182	1.18 (0.94-1.48)	0.15	1.24 (0.97-1.59)	0.09
*APOE*3	400/5,511	Reference		Reference	
*APOE*4+	125/2,051	0.83 (0.67-1.02)	0.08	0.81 (0.64-1.01)	0.06

The adjusted model controlled for age, BMI, education, income, smoking status, alcohol intake, hormone therapy, hypertension, and statin therapy at baseline.
Abbreviations: *APOE*=Apolipoprotein E; BMI=body mass index; CI=confidence interval; OR=odds ratio; T2DM=type 2 diabetes mellitus.

Supplementary Table 5. CVD incidence based on *APOE* status after adjusting for blood lipids.

CVD outcome	Cases/Total	HR (95% CI)	*p*-value
**CHD composite**			
*APOE*2+	88/883	1.23 (0.95-1.58)	0.11
*APOE*3	319/3,983	Reference	
*APOE*4+	118/1,478	1.01 (0.81-1.27)	0.90
**Stroke composite**			
*APOE*2+	65/883	0.86 (0.64-1.15)	0.30
*APOE*3	289/3,983	Reference	
*APOE*4+	105/1,478	1.12 (0.89-1.41)	0.35
**Total CVD**			
*APOE*2+	168/883	1.04 (0.87-1.25)	0.68
*APOE*3	657/3,983	Reference	
*APOE*4+	251/1,478	1.14 (0.98-1.32)	0.10

The models controlled for age, BMI, education, income, smoking status, alcohol intake, hormone therapy, hypertension, triglycerides, LDL cholesterol, and HDL cholesterol at baseline as well as T2DM incidence throughout follow-up.
Abbreviations: *APOE*=Apolipoprotein E; BMI=body mass index; CHD=coronary heart disease; CI=confidence interval; CVD=cardiovascular disease; HR=hazard ratio; T2DM=type 2 diabetes mellitus.

Supplementary Table 6. CVD incidence based on *APOE* status, including statin users at baseline (N=6,795).

		Unadjusted Model		Adjusted Model	
**CVD outcome**	**Cases/Total**	**HR (95% CI)**	***p*-value**	**HR (95% CI)**	***p*-value**
**CHD composite**					
*APOE*2+	108/912	1.03 (0.84-1.27)	0.79	1.02 (0.82-1.26)	0.88
*APOE*3	489/4,271	Reference		Reference	
*APOE*4+	171/1612	0.98 (0.82-1.16)	0.80	0.99 (0.83-1.19)	0.95
**Stroke composite**					
*APOE*2+	86/912	0.89 (0.71-1.13)	0.34	0.85 (0.67-1.09)	0.19
*APOE*3	443/4,271	Reference		Reference	
*APOE*4+	157/1,612	1.00 (0.83-1.20)	0.99	1.06 (0.88-1.28)	0.53
**Total CVD**					
*APOE*2+	213/912	0.99 (0.85-1.15)	0.89	0.95 (0.81-1.11)	0.52
*APOE*3	995/4,271	Reference		Reference	
*APOE*4+	370/1,612	1.05 (0.93-1.18)	0.41	1.10 (0.97-1.24)	0.14

The adjusted model controlled for age, BMI, education, income, smoking status, alcohol intake, hormone therapy, statin therapy, and hypertension at baseline as well as T2DM incidence throughout follow-up.
Abbreviations: *APOE*=Apolipoprotein E; BMI=body mass index; CHD=coronary heart disease; CI=confidence interval; CVD=cardiovascular disease; HR=hazard ratio; T2DM=type 2 diabetes mellitus.

Supplementary Figure 1. CVD incidence based on *APOE* status, according to baseline statin therapy (N=6,795).

CVD outcome	Baseline statin therapy	N	HR (95% CI)	*p*-value	*p*-int.
**CHD**	**No**				
**composite**	*APOE*2+	883	0.97 (0.77-1.22)	0.80	
	*APOE*3	3,983	Reference		
	*APOE*4+	1,478	1.07 (0.88-1.29)	0.51	
	**Yes**				
	*APOE*2+	29	1.70 (0.75-3.88)	0.21	0.06
	*APOE*3	288	Reference		
	*APOE*4+	134	0.48 (0.23-0.99)	0.048	0.03
**Stroke**	**No**				
**composite**	*APOE*2+	883	0.85 (0.67-1.10)	0.20	
	*APOE*3	3,983	Reference		
	*APOE*4+	1,478	1.11 (0.91-1.35)	0.30	
	**Yes**				
	*APOE*2+	29	1.00 (0.29-3.43)	1.00	0.73
	*APOE*3	288	Reference		
	*APOE*4+	134	0.65 (0.32-1.33)	0.24	0.16
**Total CVD**	**No**				
	*APOE*2+	883	0.92 (0.79-1.08)	0.33	
	*APOE*3	3,983	Reference		
	*APOE*4+	1,478	1.15 (1.01-1.31)	0.04	
	**Yes**				
	*APOE*2+	29	1.72 (0.91-3.26)	0.10	0.04
	*APOE*3	288	Reference		
	*APOE*4+	134	0.68 (0.42-1.09)	0.11	0.03

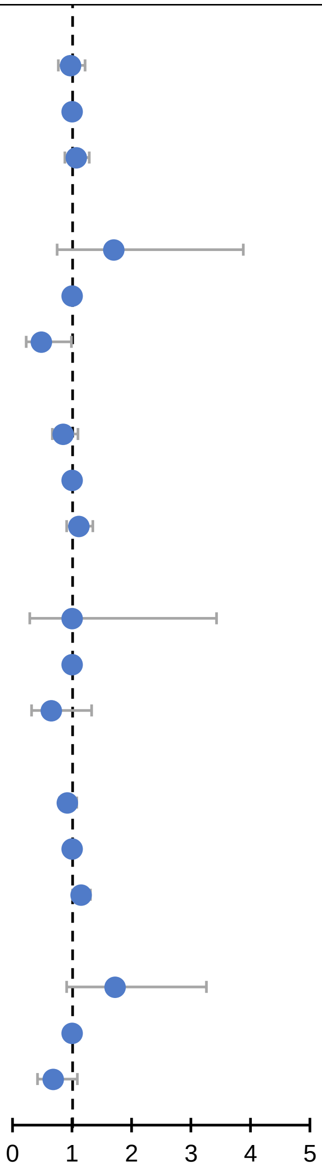

The adjusted model controlled for age, BMI, education, income, smoking status, alcohol intake, hormone therapy, and hypertension at baseline as well as T2DM incidence throughout follow-up.

Abbreviations: *APOE*=Apolipoprotein E; BMI=body mass index; CHD=coronary heart disease; CI=confidence interval; CVD=cardiovascular disease; CHD=coronary heart disease; HR=hazard ratio; T2DM=type 2 diabetes mellitus.

Supplementary Table 7. Baseline prevalence of CVD based on *APOE* status (N=8,744).

CVD outcome	Prevalent Cases/Total	Unadjusted Model OR (95% CI)	*p*-value	Adjusted Model OR (95% CI)	*p*-value
**MI**					
*APOE*2+	35/1,182	0.99 (0.68-1.43)	0.95	1.06 (0.71-1.58)	0.78
*APOE*3	165/5,511	Reference		Reference	
*APOE*4+	79/2,051	1.30 (0.99-1.71)	0.06	1.27 (0.94-1.72)	0.12
**Stroke**					
*APOE*2+	14/1,182	0.84 (0.47-1.48)	0.54	0.85 (0.46-1.59)	0.62
*APOE*3	78/5,511	Reference		Reference	
*APOE*4+	28/2,051	0.96 (0.62-1.49)	0.87	1.02 (0.64-1.63)	0.93
**Total CVD**					
*APOE*2+	199/1,182	0.96 (0.82-1.14)	0.66	1.00 (0.83-1.19)	0.96
*APOE*3	957/5,511	Reference		Reference	
*APOE*4+	351/2,051	0.98 (0.86-1.12)	0.80	0.97 (0.84-1.12)	0.68

The adjusted model controlled for age, BMI, education, income, smoking status, alcohol intake, hormone therapy, statin therapy, hypertension, and T2DM prevalence at baseline.
Abbreviations: *APOE*=Apolipoprotein E; CI=confidence interval; CVD=cardiovascular disease; MI=myocardial infarction; OR=odds ratio; T2DM=type 2 diabetes mellitus.
