# Supplementary material for: Relationships Between APOE, Type 2 Diabetes, and Cardiovascular Disease in Postmenopausal Women
Source: J Gerontol A Biol Sci Med Sci. 2024 Oct 4;80(2):glae246. doi: 10.1093/gerona/glae246 (PMC11775828; doi:10.1093/gerona/glae246)
Supplement: glae246_suppl_Supplementary_Appendix [file glae246_suppl_supplementary_appendix.pdf]

APPENDIX

WHI Investigators

Program Office: (National Heart, Lung, and Blood Institute, Bethesda, Maryland) Jacques Rossouw, Shari Ludlam, Joan McGowan, Leslie Ford, Nancy Geller

Clinical Coordinating Center: (Fred Hutchinson Cancer Research Center, Seattle, WA) Garnet L. Anderson, Ross Prentice, Charles Kooperberg, Lisa Johnson, Andrea LaCroix, Lesley Tinker, Marian Neuhouser, Susan Heckbert, Alex Reiner, Chongzhi Di, Xiaoling Song, Wayne Rosamond, Shirley Beresford, Chu Chen, Barbara Cochrane

Investigators and Academic Centers: (Brigham and Women's Hospital, Harvard Medical School, Boston, MA) JoAnn E. Manson, Shari Bassuk, Howard Sesso, Lu Wang; (MedStar Health Research Institute, Washington, DC) Barbara V. Howard; (Stanford Prevention Research Center, Stanford, CA) Marcia Stefanick, Mark Hlatky, Marco Perez, Themistocles (Tim) Assimes and Jean Tang; (The Ohio State University, Columbus, OH) Rebecca Jackson, Randall Harris, Electra Paskett, W. Jerry Mysiw, Michael Blumenfeld; (University of Arizona, Tucson/Phoenix, AZ) Cynthia A. Thomson, Tamsen Bassford, Cheryl Ritenbaugh, Zhao Chen, Marcia Ko; (University at Buffalo, Buffalo, NY) Jean Wactawski-Wende, Michael LaMonte, Amy Millen, Heather Ochs-Balcom, Christopher Andrews; (University of Florida, Gainesville/Jacksonville, FL) Marian Limacher, Michael Perri, Andrew Kaunitz, R. Stan Williams, Yvonne Brinson; (University of Iowa, Iowa City/Davenport, IA) Jennifer Robinson, Robert Wallace, James Torner, Susan Johnson, Linda Snetselaar; (University of Nevada, Reno, NV) Robert Brunner, Sandra Daughertyı; (University of Pittsburgh, Pittsburgh, PA) Lewis Kuller, Jane Cauley, N. Carole Milas; (Wake Forest University School of Medicine, Winston-Salem, NC) Sally Shumaker, Stephen Rapp, Claudine Legault, Mark Espeland, Laura Coker, Michelle Naughton

Women's Health Initiative Memory Study: (Wake Forest University School of Medicine, Winston-Salem, NC) Mark Espeland, Sally Shumaker, Stephen Rapp, Claudine Legault, Laura Coker, Michelle Naughton

Former Principal Investigators and Project Officers: (Albert Einstein College of Medicine, Bronx, NY) Sylvia Wassertheil-Smoller (Baylor College of Medicine, Houston, TX) Haleh Sangi-Haghpeykar, Aleksandar Rajkovic, Jennifer Hays, John Foreyt; (Brown University, Providence, RI) Charles B. Eaton, Annlouise R. Assaf; (Emory University, Atlanta, GA) Lawrence S. Phillips, Nelson Watts, Sally McNagny, Dallas Hall,; (Fred Hutchinson Cancer Research Center, Seattle, WA) Shirley A.A. Beresford, Maureen Henderson; (George Washington University, Washington, DC) Lisa Martin, Judith Hsia, Valery Miller; (Harbor-UCLA Research and Education Institute, Torrance, CA) Rowan Chlebowski (Kaiser Permanente Center for Health Research, Portland, OR) Erin LeBlanc, Yvonne Michael, Evelyn Whitlock, Cheryl Ritenbaugh, Barbara Valanis; (Kaiser Permanente Division of Research, Oakland, CA) Bette Caan, Robert Hiatt; (National Cancer Institute, Bethesda, MD) Carolyn Cliffordı; (Medical College of Wisconsin, Milwaukee, WI) Jane Morley Kotchen; (National Heart, Lung, and Blood Institute, Bethesda, Maryland) Linda Pottern; (Northwestern University, Chicago/Evanston, IL)

Linda Van Horn, Philip Greenland; (Rush University Medical Center, Chicago, IL) Lynda Powell, William Elliott, Henry Black; (State University of New York at Stony Brook, Stony Brook, NY) Dorothy Lane, Iris Granek; (University at Buffalo, Buffalo, NY) Maurizio Trevisan; (University of Alabama at Birmingham, Birmingham, AL) Cora E. Lewis, Albert Oberman; (University of Arizona, Tucson/Phoenix, AZ) Tamsen Bassford, Cheryl Ritenbaugh, Tom Moon; (University of California at Davis, Sacramento, CA) John Robbins; (University of California at Irvine, CA) F. Allan Hubbell, Frank Meyskens, Jr.; (University of California at Los Angeles, CA) Simin Liu, Lauren Nathan, Howard Judd[1]; (University of California at San Diego, LaJolla/Chula Vista, CA) Robert D. Langer; (University of Cincinnati, Cincinnati, OH) Michael Thomas, Margery Gass, James Liu; (University of Hawaii, Honolulu, HI) J. David Curb; (University of Massachusetts/Fallon Clinic, Worcester, MA) Judith Ockene; (University of Medicine and Dentistry of New Jersey, Newark, NJ) Norman Lasser; (University of Miami, Miami, FL) Mary Jo O'Sullivan, Marianna Baum; (University of Minnesota, Minneapolis, MN) Karen L. Margolis, Richard Grimm; (University of North Carolina, Chapel Hill, NC) Gerardo Heiss, Barbara Hulka, David Sheps; (University of Tennessee Health Science Center, Memphis, TN) Karen Johnson, William Applegate; (University of Texas Health Science Center, San Antonio, TX) Robert Brzyski, Robert Schenken; (University of Wisconsin, Madison, WI) Gloria E. Sarto, Catherine Allen[1]; (Wake Forest University School of Medicine, Winston-Salem, NC) Mara Vitolins, Denise Bonds, Electra Paskett, Greg Burke; (Wayne State University School of Medicine/Karmanos Cancer Institute, Detroit, MI) Michael S. Simon, Susan Hendrix

[1]deceased
